# Ion Release and Surface Characterization of Nanostructured Nitinol during Long-Term Testing

**DOI:** 10.3390/nano9111569

**Published:** 2019-11-05

**Authors:** Elena O. Nasakina, Maria A. Sudarchikova, Konstantin V. Sergienko, Sergey V. Konushkin, Mikhail A. Sevost’yanov

**Affiliations:** Laboratory of Durability and Plasticity of Metal and Composite Materials and Nanomaterials, Institution of Russian Academy of Sciences, A.A. Baikov Institute of Metallurgy and Material Science RAS (IMET RAS), Leninsky Prospect 49, 119991 Moscow, Russia; bloodymaria@list.ru (M.A.S.); shulf@yandex.ru (K.V.S.); venev.55@mail.ru (S.V.K.); cmakp@mail.ru (M.A.S.)

**Keywords:** alloy, nickel, titanium, pitting corrosion, repassivation

## Abstract

The corrosion resistance of nanostructured nitinol (NiTi) was investigated using long-term tests in solutions simulating physiological fluids at static conditions, reflecting the material structure and metal concentration in the solutions. Mechanical polishing reduced the ion release by a factor of two to three, whereas annealing deteriorated the corrosion resistance. The depassivation and repassivation of nitinol surfaces were considered. We found that nanostructured nitinol might increase the corrosion leaching of titanium into solutions, although the nickel release decreased. Metal dissolution did not occur in the alkaline environment or artificial plasma. A Ni-free surface with a protective 25 nm-thick titanium oxide film resulted from soaking mechanically treated samples of the NiTi wire in a saline solution for two years under static conditions. Hence, the medical application of nanostructured NiTi, such as for the production of medical devices and implants such as stents, shows potential compared with microstructured NiTi.

## 1. Introduction

Nitinol (NiTi) is an alloy characterized by high mechanical biocompatibility, including super elasticity and compliance with the delay law, all of which enable integration with living tissue [1,2,3,4,5,6]. NiTi also has shape memory effects that allow its application in the development of intelligent systems with programmable shapes.

However, the highly toxic Ni content of the alloy is capable of causing DNA damage, inflammation, formation of O radicals, tissue death around NiTi implants, tumor growth, allergies, and genetic mutations, and this has limited its application in medical devices [7,8,9,10,11,12]. No exact information is currently available about the tolerance limit of Ni content in an in vivo environment. However, concentrations of nickel ions significantly suppressed cell proliferation even at 25 μM after three days [7]. Growth inhibition of smooth muscle cells became significant when the nickel concentration was above 9 ppm [8]. A threshold value of 30 ppm was needed to trigger a cytotoxic response during in vitro experiments [9]. After seven days, necrosis spread 1 mm around the implant and nickel concentrations reached 48 μg/g near the implants [10]. The Ni dispersion area was consistent with the inflammatory area and the degree of tissue damage was closely related to the dissolved Ni concentration [11]. The possibility of destruction by corrosion under variable dynamic loads limits the lifetime of devices fabricated from the alloy.

The electrochemical properties of NiTi are reported to be similar to those of corrosion-resistant Ti. However, various researchers have noted that the electrochemical properties of NiTi in medical applications range from poor to good [1,13,14,15,16,17,18,19,20,21,22,23]. Widely varying concentrations (1–100 mg/L) and durations (hours to months) of Ni ion release have also been reported for microstructured NiTi in physiological or saline environments [22,23,24,25,26,27,28,29,30,31,32,33]. Therefore, both positive and negative results have been reported in the above studies, as well as in vivo and in vitro biological studies of nitinol [1,8,22,32,33,34,35,36,37,38,39,40,41], leading to ambiguous and conflicting conclusions regarding its utility. However, an observation that has been common to all NiTi investigations is that the surface conditions of the alloy strongly affect its resistance to dissolution. Specifically, the corrosion resistance of the material has been determined to be better when the surface oxide layer is less damaged and contaminated.

Heat treatment of nitinol at 300–1000 °C is required to stabilize the relevant mechanical properties of the material and to shape a product; however, this may also adversely affect the corrosion resistance [16,19,26,42,43,44,45,46]. Heating causes the formation of a thick and uneven surface layer consisting of a mixture of Ni and Ti oxides. This oxide layer is extremely unstable under applied loads and contributes to the diffusion of nickel into the environment, which indicates the necessity of additional processing.

A wide range of methods are available for facilitating the formation of an improved homogeneous and naturally occurring titanium oxide surface layer with a minimum Ni content and a film thickness of up to several hundred nanometers. These methods include chemical passivation, anodizing, laser oxidation, oxidation in boiling water and autoclaves, electropolishing, and deposition [16,18,19,25,26,27,29,42,43,44,45,46,47,48,49,50,51,52,53,54,55,56,57,58,59,60,61,62,63,64,65,66,67,68,69,70,71,72,73,74,75,76,77,78]. However, these methods cause problems such as reduced biocompatibility, reduced corrosion resistance under cyclic thermal and mechanical loads, and mechanical property degradation [17,28,41,47,48,49,50,56]. Some of these treatment methods are not applicable to products with complex configurations, are expensive, or may induce heterogenous residual stress fields [16]. Mechanical surface treatment does not remove Ni from the surface and does not significantly increase the corrosion resistance and biocompatibility of the material [16,26,27,45].

Although improvements to the mechanical properties of NiTi are not needed, its corrosion resistance and biocompatibility urgently require enhancement. Managing the properties of NiTi and NiTi-based alloys to enable their processing and use in the fabrication of medical implants and equipment has been extremely difficult. While NiTi has been successfully applied in medical devices such as stents, the properties of prostheses created from the material require improvement. The creation of nanostructures in the material shows promise for addressing some of the challenges. In this study, we investigated the corrosion resistance and related surface morphology and surface composition changes of nanostructured NiTi under static conditions through long-term testing in different solutions.

## 2. Materials and Methods

Nanostructured nitinol wires (IMET RAS; Moscow, Russia) were investigated. They were obtained according to the scheme given below. We subject 56 wt % Ni–44 wt % Ti mixture to multistage re-melting in a vacuum furnace under an argon atmosphere. Ingots weighing less than 400 g were subjected to homogeneous annealing at 750–1000 °C for 10 h to stabilize the grain structure. After, the ingots were sequentially converted by stepwise hot rolling at 700–750 °C and rotary forging at 750–1000 °C into bars of up to 4 mm in diameter. From the bars, wires, 0.4–0.28 mm in diameter, were obtained by multiple stepwise hot drawing through synthetic diamond dies. Between all the steps, intermediate heat processing was also performed.

The surface of the samples was subsequently mechanically treated along the axis of the samples using sandpaper (abrasive grain sizes from 180 to 1000 grit) and diamond paste (3 µm followed by 1 µm) to a mirror finish. This process resulted in a reduction of the sample diameter by up to 10 µm compared to the as-received samples (after production). The as-received and mechanically treated wires were annealed at 450 °C for 15 min in air using a muffle furnace (LOIP LF series, model 7/13-G2, Moscow, Russia). The wire diameter was unchanged during annealing.

The characteristic grain type and size of the wire samples were determined by transmission electron microscopy (TEM; TECNAI 12, FEI Company, Hillsboro, Oregon USA). The samples were prepared using an ion etching apparatus (Gatan 691, Gatan Inc., Pleasanton, CA, USA).

The phase occurrence of the samples was determined by X-ray diffraction (XRD; Ultima IV, Rigaku Co., Woodlands, TX, USA) using Cu Kα radiation with a graphite monochromator, vertical goniometer, and rapid semiconductor detector (D/teX). The Bragg–Brentano method was employed. Phase analysis was conducted using the PDXL program complex (Rigaku Co., Woodlands, TX, USA) and ICDD database.

The morphology and sub-surface elemental composition of the samples were investigated using scanning electron microscopy (SEM; TESCAN VEGA II SBU, TESCAN, Brno, Czech Republic) with an attached energy-dispersive X-ray spectroscopy (EDS) module (INCA Energy). Auger electron spectroscopy (JAMP-9500F, JEOL Co., Tokyo, Japan) combined with ion beam etching during argon bombardment at an angle of 30° was also employed. All nitinol samples were ultrasonically cleaned in alcohol immediately before analysis, except for samples immersed in solutions. The latter were not cleaned to prevent the washing off of any possible sediment.

For microstructural analysis, the samples of nanostructured nitinol wires used for the actual tests were prepared by 2–3 min of pre-etching using a mixture of HF (1 mL), HNO_3_ (2 mL), and water (47 mL), followed by rinsing multiple times with distilled water and then air drying. A metallographic optical microscope (Axiovert 40 MAT, Carl Zeiss, Oberkochen, Germany) with digital image processing capability was used to image the samples.

Since pH values ranging from ~1 to 9 are found in the human body (for example, pH = 1.05 in a duodenal ulcer, 1.53–1.67 in gastric acid, 3.8–4 close to the intestinal canal wall, 7.34–7.43 in blood; and 8.5–9 in the large intestine), we decided to conduct tests at several pH values. We used seven solutions: four standard buffer solutions (prepared from pre-made buffer kits (Merck) with pH values of 1.68–4.01, 9.18), HCl (aq., 0.0275 M, pH 1.56), NaCl (aq., 0.9 wt %, pH 6.3), and artificial plasma (pH 7.36) (Table 1) [16,17,20,23,26,27,29,30,46,49,78].

Four types of samples of the nanostructured NiTi wires corresponding to four different states during the treatment—as-received (Sample 1), after annealing (Sample 2, at 450 °C for 15 min), after mechanical treatment (Sample 3, using sandpaper with abrasive grain sizes from 180 to 1000 grit and diamond paste), and after mechanical treatment and subsequent annealing (Sample 4)—were used for the tests, with each wire weighing 32.6 g. Wire samples of each type were placed in 100 mL of the different test solutions contained in flat-bottomed flasks composed of thermal laboratory glass (for the neutral and acidic solutions) and polypropylene (for the alkaline solution). The flasks were corked tightly and stored in the dark in intervals between sampling (Table 2). The solutions were analyzed by inductively coupled plasma atomic emission spectroscopy (ICP-AES; ULTIMA 2, HORIBA Jobin Yvon, Kyoto, Japan).

## 3. Results

### 3.1. Structure Analysis Before Corrosion

**Figure 1:**
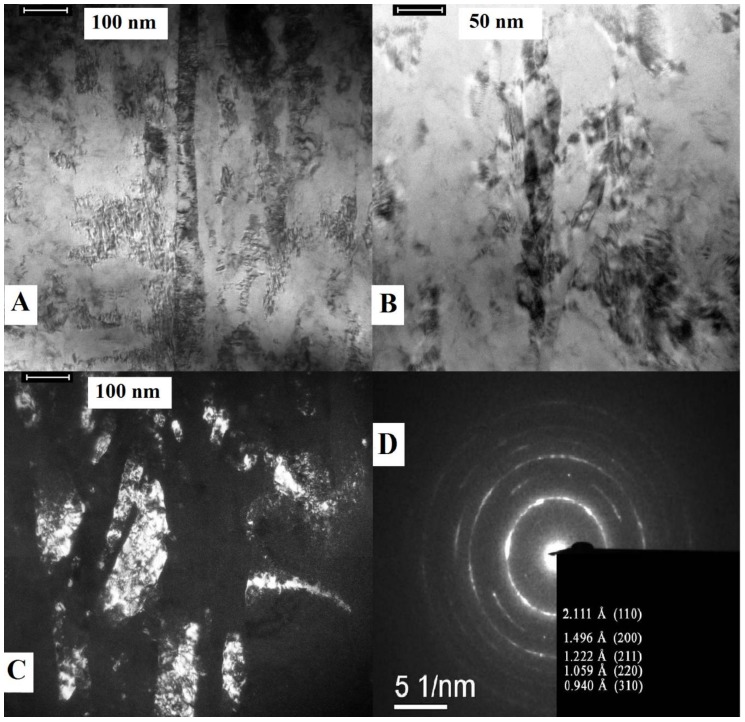
(**A,B**) Bright-field and (**C**) dark-field TEM images of the grain structure and (**D**) microdiffraction pattern of the nitinol wire before treatments. Bright-field images illustrate coaxial elongated grains; dark-field image illustrates more randomly spaced grains and its etched fragments.

The bright- and dark-field TEM images of the nanostructured NiTi wires (Figure 1) showed that the alloy grains occurred as nanofibers 30–70 nm in diameter and several micrometers long, with the grains extending along the wire axis. The microdiffraction, EDS, XRD, and microstructural analyses revealed that the main component of NiTi was a B2-phase TiNi, with flecks of Ti_2_Ni intermetallic compounds [79]. The composition was found to be unchanged by the treatment processes.

**Figure 2:**
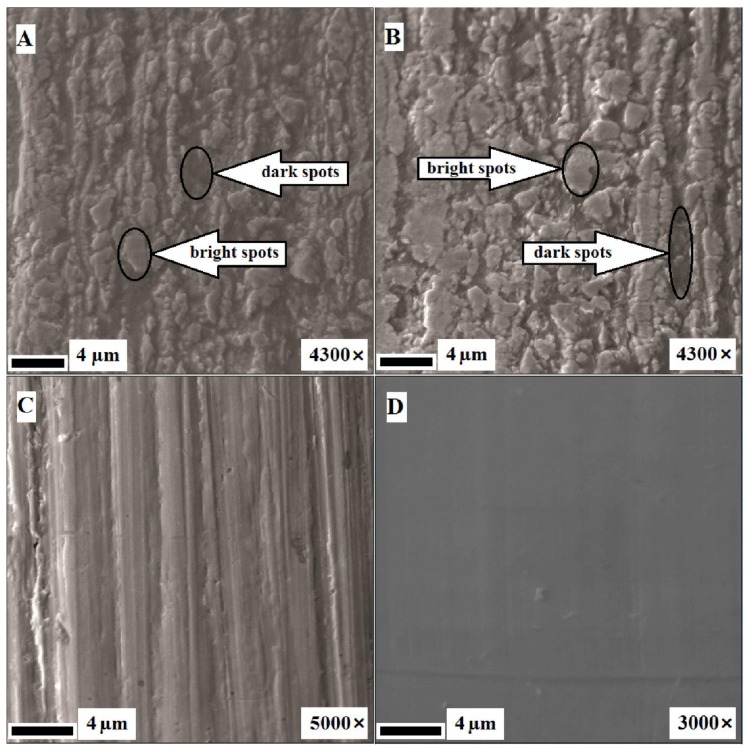
SEM images of the NiTi wire surface before immersion testing: (**A**) as-received (before processing); (**B**) after annealing at 450 °C for 15 min; (**C**) after mechanical treatment to a mirror finish in the direction of the long axis of the samples using sandpaper (abrasive grain sizes from 180 to 1000 grit) and diamond paste (3 µm followed by 1 µm); and (**D**) mechanically treated and annealed sample. “Dark spots” and “bright spots” indicate different surface areas for additional composition analysis.

The SEM images in Figure 2A show that the surface of the NiTi wires before treatment (as-received) was non-uniform, with the presence of light and dark areas of varying contrast, as well as obvious roughness and drawing defects, including fluted reliefs along the drawing axis and dimples caused by the press of solid particles into the surface. After annealing (Figure 2B), the surface remained mostly the same, the only difference being the presence of a higher proportion of bright spots. After mechanical treatment (Figure 2C), almost all the defects and roughness were removed, with no spots observed. Only the machining marks were present, which existed in the form of grooves with a depth and width of 1 µm. Subsequent annealing (Figure 2D) smoothed the surface.

**Figure 3:**
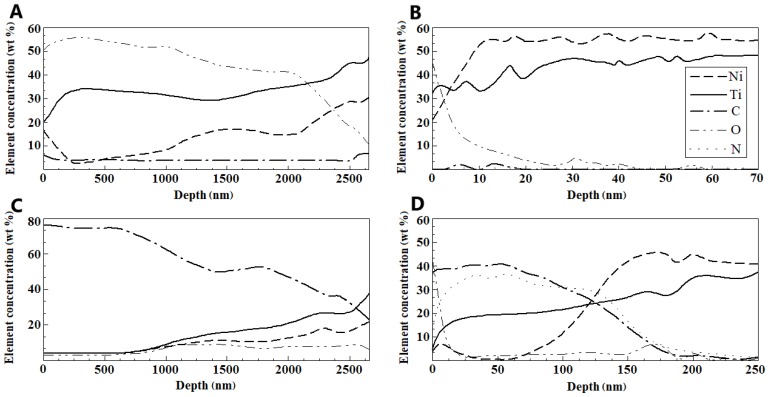
NiTi wire surface composition according to Auger electron spectroscopy before immersion tests: elemental depth profiles of (**A**) bright region (identified in Figure 2A,B) and (**B**) dark region (identified in Figure 2A,B) of the as-received sample and the sample annealed at 450 °C for 15 min; surfaces of samples (**C**) mechanically treated using sandpaper (abrasive grain sizes from 180 to 1000 grit) and diamond paste (3 µm followed by 1 µm) and (**D**) mechanically treated and annealed.

The bright and dark spots on the surface had different compositions, as indicated by the Auger electron spectroscopy results (Figure 3A,B). High concentrations of titanium oxide were observed in the bright spots (Figure 3A), whereas the dark spots were rich in carbon (Figure 3D). Both regions were ~3 µm thick and did not overlap. The annealing slightly increased the proportion of the surface oxide (Figure 2A,B). The composition of the mechanically treated surface was uniform (Figure 3C); the wire was covered by an oxide layer less than 10 nm thick. Subsequent annealing contributed (Figure 3D) to the formation of an oxide–nitride layer up to 80–150 nm deep. Nickel was not completely removed from both surfaces.

### 3.2. Surface Analysis After Corrosion

**Figure 4:**
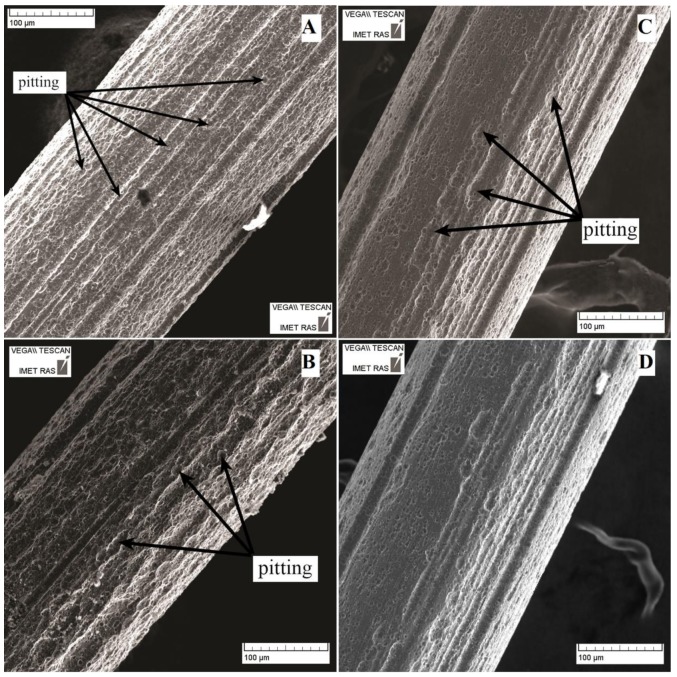
SEM image of the NiTi wire surface after two years of immersion testing in a buffer solution at pH 1.68 (Solution 1, Table 1): (**A**) as-received sample; (**B**) sample annealed at 450 °C for 15 min; (**C**) sample mechanically treated in the direction of the long axis using diamond paste (3 µm followed by 1 µm) sample; and (**D**) sample mechanically treated and annealed.

The results of the surface SEM investigations showed that the annealed samples suffered the greatest corrosion damage after exposure to the different solutions (Figure 4). The least damaged samples were those that were mechanically treated (both annealed and not). The non-uniform surface corrosion resulted in the formation of deep pores (pitting). The SEM images suggested that the pitting corrosion damage was due to the drawing defects.

**Figure 5:**
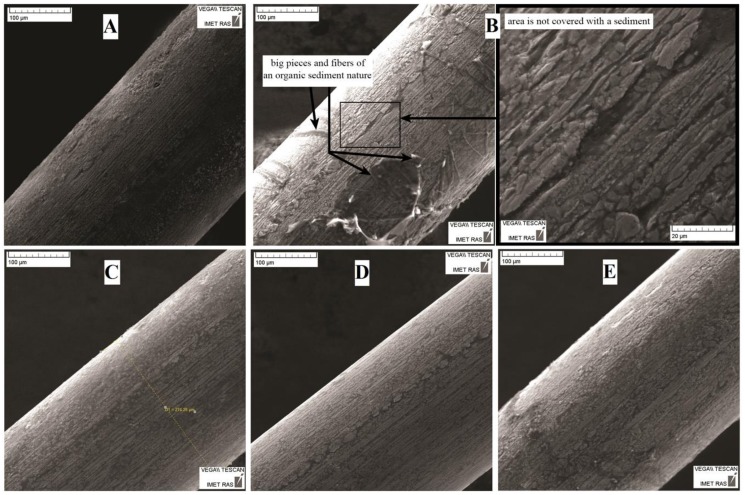
SEM images of the as-received NiTi sample surface after two years of immersion in various solutions: (**A**) buffer at pH 3.56 (Solution 2; Table 1); (**B**) buffer at pH 4.01 (Solution 3, Table 1); (**C**) 0.9 wt % NaCl (Solution 4, Table 1); (**D**) pH 9.18 (Solution 5, Table 1); and (**E**) HCl (pH 1.56).

Visual inspection after two years of storage (Figure 4A and Figure 5; Table 3) suggested that the strongest corrosion occurred in the pH 1.68 solution. Traces of corrosion in the form of loosening surfaces were observed in the NiTi wire samples exposed to the weakly acidic solution of pH 3.56 (Figure 5A; Table 3). No surface damage was observed in the samples exposed to the pH 4.01 solution, although organic sediments partially covered the samples (Figure 5B; Table 3). In NaCl (Figure 5C; Table 3), the wire samples were covered by a homogeneous surface layer that was smoother than the surface of the as-received samples, and no evidence of pitting was observed. We observed the same for the wire samples exposed to the artificial plasma. We suggest that this layer was produced by repassivation of the damaged surface. No changes were observed in the diameter and surface structure of the wire samples exposed to the alkaline solution (Figure 5D; Table 3). No release of metallic ions was observed from the NiTi wire samples immersed in the pH 9.18 solution, as substantiated by the unchanged appearance of the samples after the immersion.

**Figure 6:**
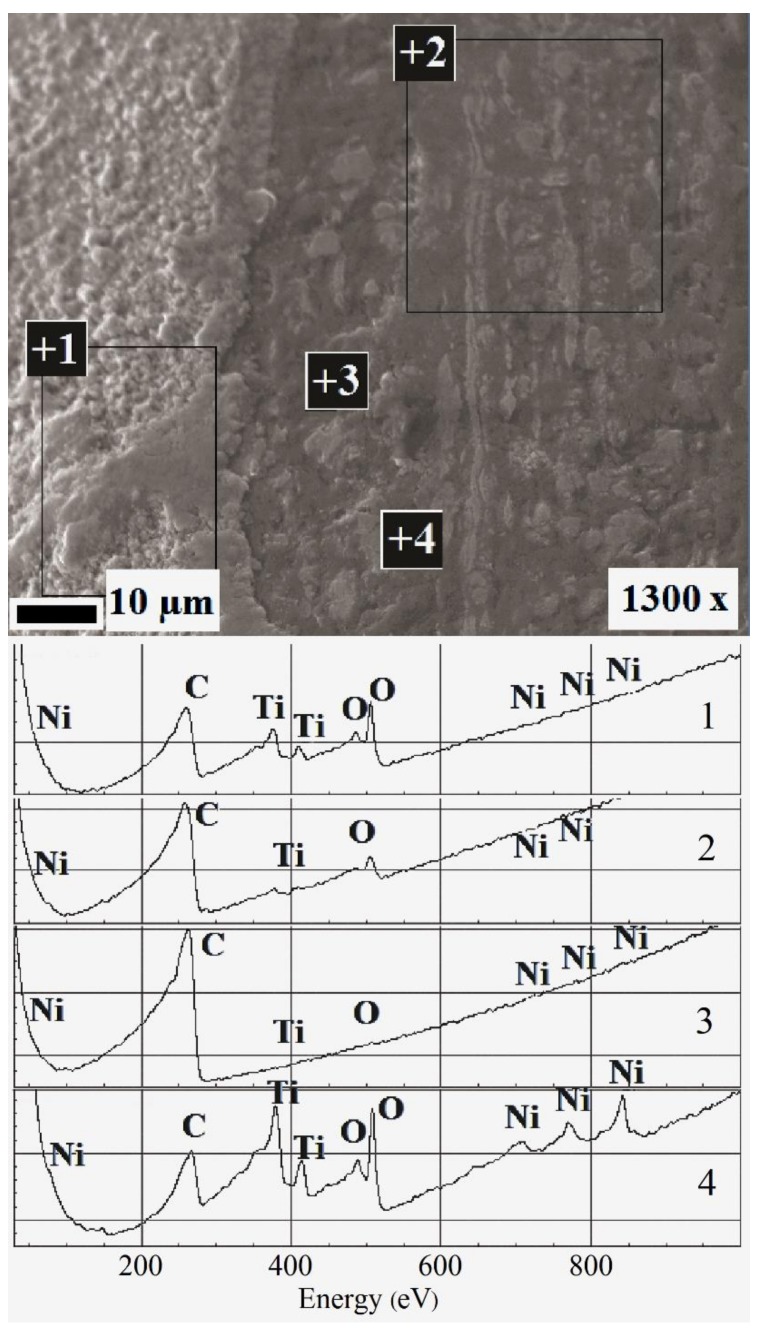
Surface structure of as-received nitinol after immersion in 0.9 wt % NaCl solution (Solution 4, Table 1) for two years. The Auger electron spectra (lower) were collected for the corresponding surface analysis areas (upper).

The surface compositions of both the as-received and annealed TiNi wire samples were observed to be unchanged by the long-term exposure to the different solutions. The samples still contained the alternating dark C-containing areas with low Ni contents and light areas of titanium oxide with Ni inclusions (Figure 6). The auger electron spectroscopy results showed the depths of these areas to be greater than 1 µm. However, long-term immersion in a Cl-containing solution increased the oxide proportion on the surface. This is consistent with previously reported findings [14,20,23,24,27,33].

**Figure 7:**
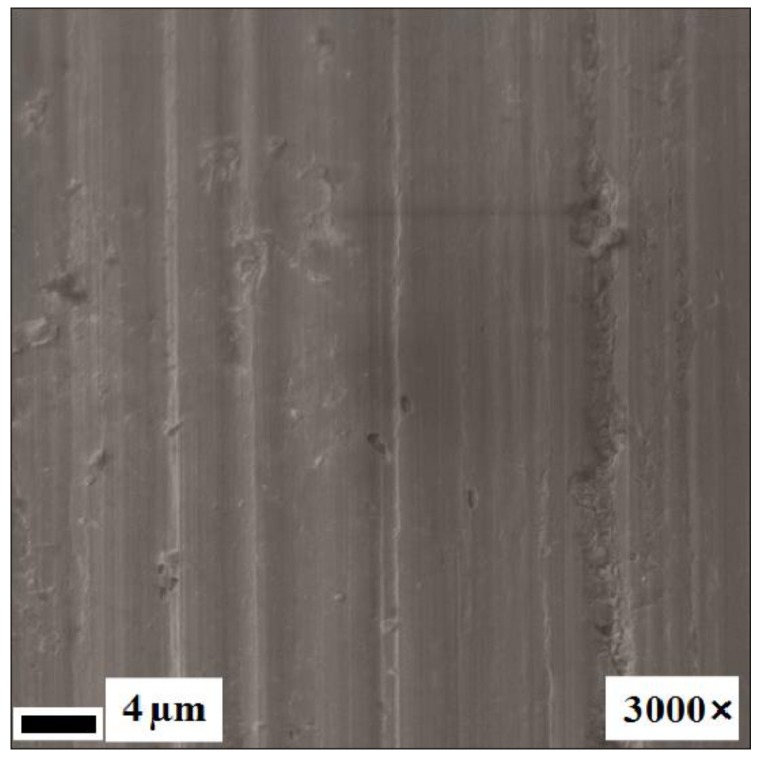
SEM image of the NiTi wire surface (mechanically treated in the direction of the long axis of the samples using diamond paste (3 µm followed by 1 µm)) after immersion for two years in Solution 2 (Table 1) at pH 3.56.

**Figure 8:**
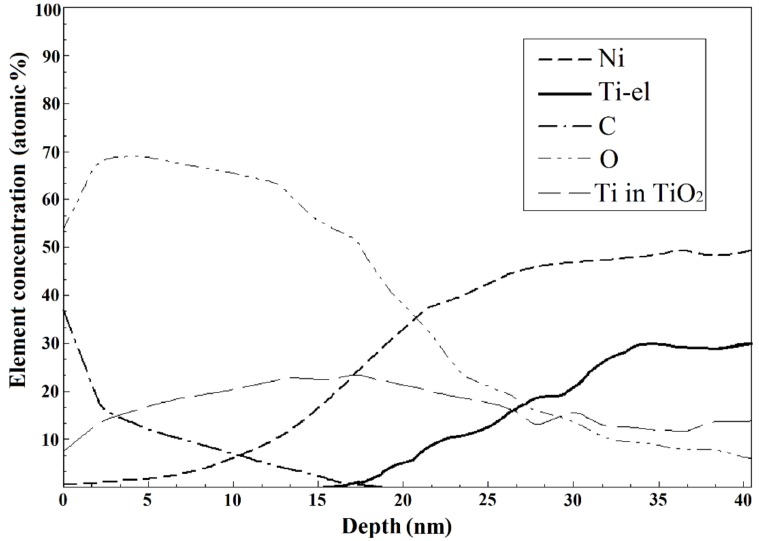
Composition of the NiTi wire surface mechanically treated in the direction of the long axis of the samples using diamond paste (3 µm followed by 1 µm) after immersion for two years in 0.9 wt % NaCl (Solution 4, Table 1), as determined by Auger spectroscopy. “Ti in TiO_2_” and “Ti-el” mean bonded and elemental forms of Ti, respectively.

The surfaces of the mechanically treated samples after immersion for two years are shown in Figure 4C and Figure 7. Pitting was only observed in the pH 1.68 buffer solution. The surface oxide layer of the mechanically treated samples was found to extend to a maximum depth of ~25 nm after immersion in the neutral solutions (Table 4). The relative proportions of the bonded and elemental forms of Ti in the samples immersed in NaCl solution were determined from the Auger data, which also revealed that no elemental Ti was observed until a depth of 17 nm (Figure 8). No Ni was observed on the corroded surface of the present samples immersed in NaCl. The carbon concentration in the surface layer was observed to decrease sharply from a high level on the surface to zero at ~15 nm. This suggests mechanically induced surface contamination.

**Figure 9:**
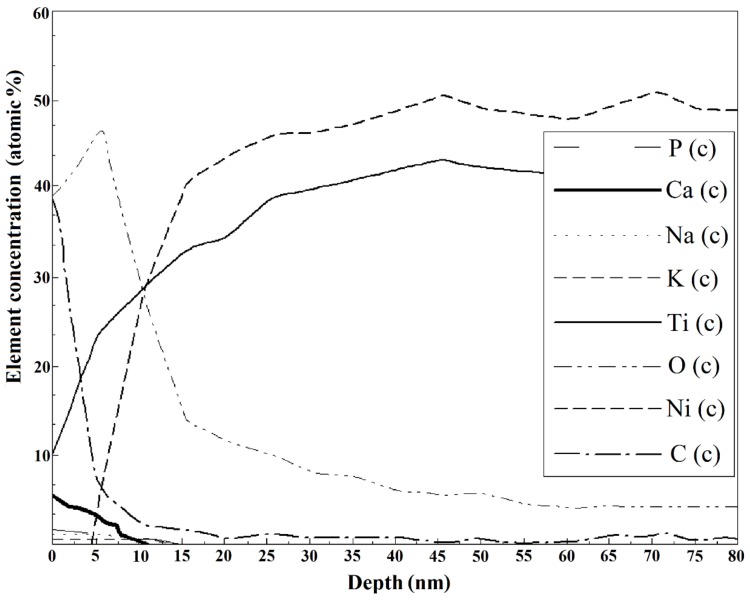
Composition of the NiTi wire surface mechanically treated in the direction of the long axis of the samples using diamond paste (3 µm followed by 1 µm) after immersion for two years in artificial plasma (Solution 6, Table 1) as determined by Auger spectroscopy.

The surface of the wire samples immersed in artificial plasma was observed to contain Ca, P, Na, and K (Figure 9), which can be attributed to inorganic sedimentation.

### 3.3. Metallic Ion Release in Solutions

**Figure 10:**
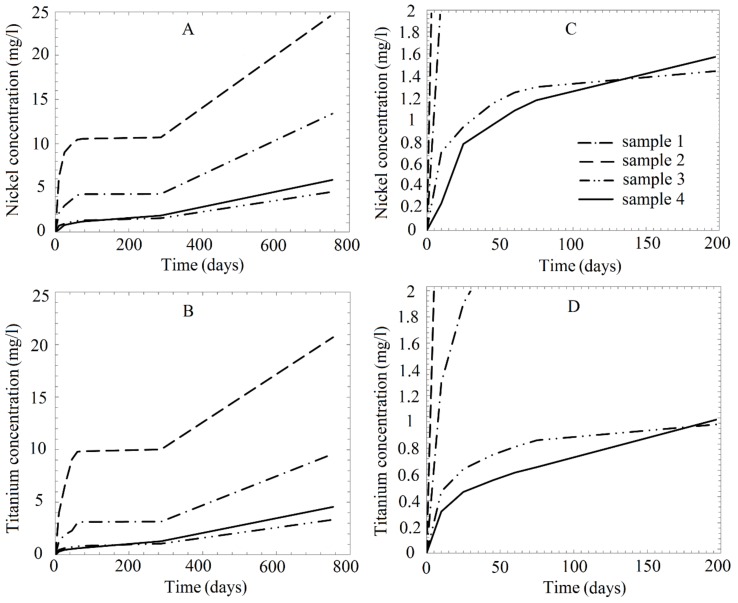
Change in the concentration of ions over time as each type of NiTi wire sample (Sample 1 – as-received, Sample 2 – annealed at 450 °C for 15 min, Sample 3 – mechanically treated in the direction of the long axis of the samples using diamond paste (3 µm followed by 1 µm), and Sample 4 – mechanically treated and annealed) was immersed in a buffer solution at pH 1.68 (Solution 1, Table 1): (**A,C**) nickel and (**B,D**) titanium.

**Figure 11:**
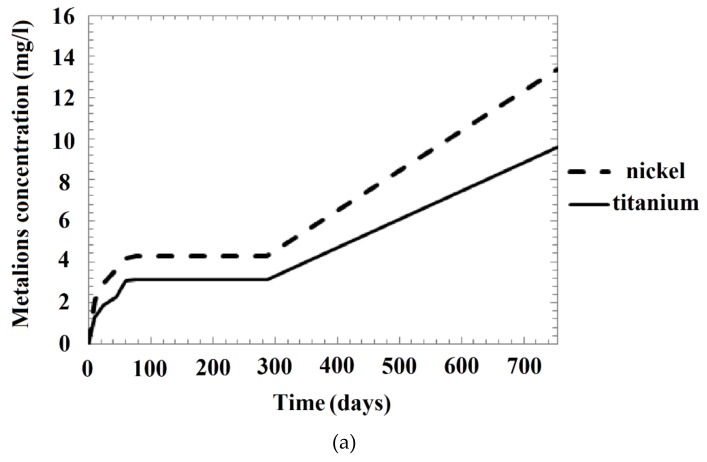

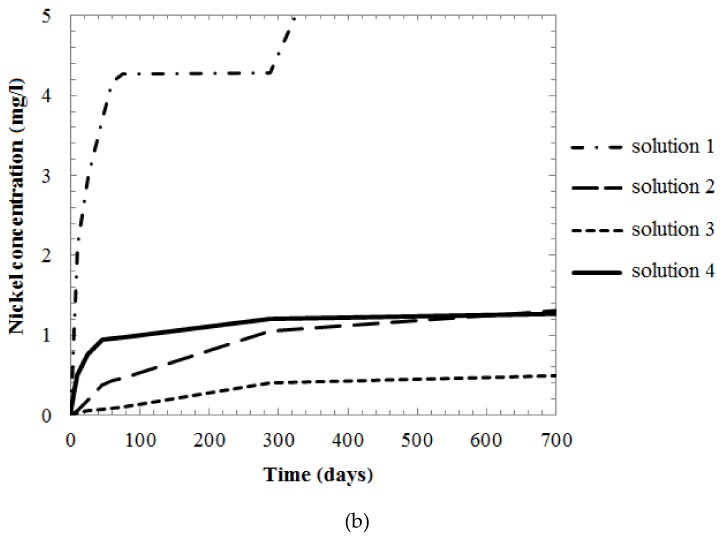
Changes in the concentration of ions dissolved from the as-received nitinol sample with time during immersion for 2 years: (**a**) nickel and titanium ion concentrations in a buffer solution at pH 1.68 (Solution 1, Table 1); and (**b**) nickel ion concentration in solutions with various acidities (Solution 1 – pH 1.68 buffer, Solutions 2 – pH 3.56 buffer, Solution 3 – pH 4.01 buffer, Solution 4 – NaCl, Table 1).

The results of long-term examinations (two years) of metallic ion release from the nanostructured NiTi samples placed in acid solutions and normal saline are presented in Table 2 and Figure 10 and Figure 11. The results indicate the occurrence of slight corrosion in the solutions. In the case of the samples placed in alkaline solution, as noted earlier, and in artificial plasma, no release of metal into the solution was observed during the test period. Therefore, no corresponding marks are provided in Table 2 and in the figures.

The annealed samples were the most prone to corrosion (Figure 10): their nickel and titanium release was found to be three-times higher than that of the untreated samples. The rates of change of the Ni and Ti concentrations in solution over time were almost the same. Expectedly, the corrosion resistance of the mechanically treated NiTi samples was significantly higher than that of the as-received samples. However, the wire diameters changed disproportionately with the ion concentrations in the solutions (Table 3). For example, the diameters of the as-received and annealed wires were similar after storage in the pH 1.68 solution.

The release of ions was continuous during the two-year duration of the experiment; the concentrations of both nickel and titanium ions in solution increased with time (Figure 11a). However, the variation in the concentration of the Ni ions depended on the solution type, as shown in Figure 11b. The figure shows that the gradient of the Ni ion concentration in NaCl decreases smoothly with time until a constant concentration is attained. The Ni ion concentration in the weakly acidic solution was initially lower than that in the neutral chloride-containing solution, but increased after approximately 600 days. In the strongly acidic solution (pH 1.68), the sharp increase in the ion concentration resumed after a temporary plateau.

The buffer solutions used in the present study are not representative of typical physiological liquids and were only used to create environments with different pH values. Their effects on NiTi were thus compared with that of 0.0275 M hydrochloric acid. The Ni and Ti concentrations in the HCl solution with as-received NiTi sample after 10 days were 1.94 and 0.515 mg/L, respectively (Figure 10A, Table 2).

## 4. Discussion

### 4.1. Structure Analysis Before and After Corrosion

The presence of a thick mixed-composition layer on the surface of the as-received wire (Figure 3) was presumed to be due to the prolonged intermediate heat treatments during wire production [16,19,26,42,43,44,45,46]. It is thus unlikely that annealing the wire for 15 min would have significantly affected the surface composition. The abundance of carbon in the wire samples (Figure 3B) is likely due to the use of graphite-containing lubricants during the wire drawing process, with the lubricant remaining on the wire surface being fixated by the annealing. The presence of impurities on the surface of a NiTi sample after several cycles of treatment involving the use of a lubricant containing these elements was previously reported [25]. Our data fully agree with the data reported in previous studies [79].

The removal of the surface impurities and defects, reduction of the contact area, and formation of a more uniform passivating film (i.e., the observed homogeneous ~10 nm-thick oxide layer (Figure 2C and Figure 3C), or the previously reported ~4 nm-thick oxide layer on polished samples [24]) that result from the mechanical treatment evidently increase the corrosion resistance of the material. However, the presence any Ni on the surface, even if only a small amount, is undesirable. A thin passivating oxide layer is preferable because it is more flexible and can adapt to applied loads [42], although the layer must be thicker than 100 nm to act as an anticorrosive barrier [25]. Thus, the thin surface oxide layer formed in this study does not offer optimal protection against corrosion.

The surface composition of the mechanically treated samples after immersion for two years (Figure 8) agrees with the observations in Hu T. et al. [24], who also reported that the surface layer of polished samples immersed in NaCl solutions, as noted here, contains only titanium oxides, with Ti (corresponding to the NiTi phase) appearing and then increasing with increasing depth, gradually becoming more dominant. No Ni was observed on the corroded surface immersed in NaCl because of the release of Ni ions into the corroding solution, whereas Ti remained on the wire surface and reacted with the dissolved O in the solution to form an oxide layer in the damaged areas.

Inorganic sedimentation (Figure 9) can result in the prevention of the dissolution of Ni and Ti in an artificial plasma solution [14,23,26,67,80]. This inorganic-sedimentation layer may provide additional protection against corrosion.

Previous studies have shown that the exposure of NiTi to a hostile environment for an extended period leads to the formation of a thickened oxide layer [24]. This was substantiated by our reported observations (Figure 8 and Table 4).

### 4.2. Metallic Ion Release in Solutions

Notably, during the initial period of the studies, the release of ions from sample 3 was higher than from sample 4, and after 140–180 days, the opposite was observed (Figure 10). We assumed that, at first, the thicker surface layer of the sample after mechanical surface treatment and annealing acted as a better barrier against the diffusion of ions into the solution; however, an increase in the heterogeneity of the outer oxide layer as a result of heat treatment did not slow the leaching of elements as effectively as the more uniform passive film originally obtained by mechanical surface treatment [79].

Based on previous reports, we attribute the plateau in the concentration curve (Figure 11) to the sequential destruction and renewal of the protective oxide layer (i.e., depassivation and repassivation) in the defect areas [17,24,26,81]. We propose that these processes occurred in all solutions, with the concentration variation being less noticeable compared to the highly acidic solution.

Our observations of the rate of concentration change (Figure 11) and the surface conditions (Figure 5) for the most acidic solution are similar to previous reports for NaCl [24]. Specifically, after the formation of pitting pores, which grow into larger holes surrounded by new pitting pores, the release of Ni increases; however, the oxide layer then decreases the corrosion speed by sealing the pores.

Previous studies found that the maximum rate of Ni ions release occurred at the beginning of the test before the rate reduced dramatically (for the period from 8 to 14 days to several months) due to the thickening of the oxide layer, which serves as a protective barrier [13,14,15,16,17,18,19,20,21,22,23,24,27,33]. From evaluations of the repassivation processes that occur under static and dynamic conditions and after surface damage, nitinol is resistant to the destruction of the passive layer under dynamic loading, and has a greater tendency for repassivation compared to 316L stainless steel [13,81,82,83]. Samples of NiTi wires of different compositions have also been observed to develop passivation layers with the same composition after immersion in solutions, with the layers consisting of Ni-containing Ti oxides [14,20,23]. The possibility of repassivation was also noted in these previous works.

Each sample type in the present study exhibited the highest ion release in the most acidic environment (Figure 11b), with the ion release decreasing with increasing pH value, and then beginning to increase again in the neutral NaCl. Under all the considered test conditions, more Ni ions were released than Ti ions (Figure 10 and Figure 11a).

We found that exposure to a saline solution leads to a high level of metal ion release. During the early stage of the test, the ion release was higher than those in pH 4.01 and 3.56 solutions, with the metal ion concentration in the saline solution remaining higher than in the pH 4.01 solution after the first 600 days (Figure 4B; Table 2). The saline solution is a sufficiently concentrated source of chloride ions, which have higher affinity for metals than O and thus replace O in the protective oxide surface layer, resulting in depassivation and pitting [84,85]. The speed of the NiTi corrosion process was observed to increase with increasing chloride concentration in the saline solution, being strongly dependent on the surface conditions [86]. It was noted that NiTi corroded via pitting at an appropriate chloride concentration across the entire pH range and the pitting potential did not vary with the Ni ion concentration [87].

Withalmetallic ion release and surface damage increased with increasing acidity (Figure 4A, Figure 5A,B and Figure 10; Figure 11b; Table 2), which indicates an increasingly hostile environment. Whereas the findings of some studies suggest that acidity has a negligible impact on the corrosion resistance of NiTi [88,89], others have linked an increase in the corrosion rate, observed as an increase in the rate of Ni ion release, to decreasing pH [14,23,31]. Another study [87] showed that the breakdown potential in an alkaline environment was 100 mV higher than in an acidic environment, where it was 100–200 mV higher than in a neutral environment (attributed to the general etching of the alloy surface in an acidic solution). This agrees with our observations that no corrosion occurs in an alkaline medium (Figure 5D; Table 3).

When comparing the effect of buffer solutions and hydrochloric acid on nitinol, we noted that the dissolved Ni content after a short exposure to the pH 1.56 HCl was comparable with that in the pH 1.68 buffer solution (Figure 10A; Table 2). However, the titanium concentration in the buffer solution was two-times higher than that in the HCl (Figure 10B; Table 2). Expectedly, the pH of the HCl solution was unstable and increased to pH 2.00 after two years of carrying the NiTi wires. The surface of the wires after long-term exposure to HCl (Figure 5E) resembled that of the wires immersed in NaCl solution (Figure 5C), although the former suffered more corrosion damage. In contradistinction to the samples exposed to the highly acidic buffer solution with the most serious pitting surface damage samples after exposure to HCl were covered with a rough and presumably oxide layer. The changes in the wire diameter in HCl (Table 3) were also significantly less than those in the acidic pH 1.68 buffer solution.

From the observed ratios between the amounts of released Ni and Ti ions, the wire diameter changes (Table 3), and the surface morphology changes (Figure 4A; Figure 5C,E) in this study, we inferred that the behaviors of NiTi in acidic (HCl) and neutral (NaCl and plasma) chloride-containing environments are more similar than those in the two acidic solutions (HCl and pH 1.68 buffer solution). The surface of the NiTi wire samples in HCl was observed to be covered with a protective layer. We assumed that the amounts of Ni ions released in the two acidic environments are comparable, whereas more titanium will be consumed in the formation of a protective oxide layer, as in neutral media.

However, as noted earlier, the solutions and conditions considered in the present study are not representative of physiological conditions and were only used to model some corrosion parameters. Although this enabled the acquisition of some information about the typical behavior of NiTi, further biological investigations are required to properly predict the behavior of the alloy in physiological systems.

### 4.3. Effect of Nanostructure and Treatment on Corrosion Behavior

Polycrystalline NiTi is a self-passivated material, like Ti or stainless steel. The grain boundaries contain many O atoms, which create a nonstoichiometric oxide film around each grain through their reaction with Ti (TiII, TiIII, and TiIV species). This stable surface layer protects each grain and the base material against corrosion [1,24,90]. Therefore, increasing the number of grain boundaries in the volume of the alloy could increase its corrosion resistance and biocompatibility. However, the high density of intergranular surface defects could lead to a poor corrosion performance since corrosion attack typically initiates at surface heterogeneities [91]. This has been little studied.

Here, the concentrations of the Ni released from the nanostructured NiTi into the different solutions (Table 2) were lower than the previously reported average values for microstructured NiTi [22,23,24,25,26,27,28,29,30,31,32,33]. Examples of the reported nickel release in solutions similar in composition and/or acidity to the solutions used in this study are summarized in Table 5. These values are widely different, even within one study.

We unexpectedly found that Ti ions were released in all the solutions considered in this study (Figure 10 and Figure 11). The only exceptions were the cases of the mechanically treated samples in the neutral and pH 4.01 solutions. - In case of other samples and solutions, it can be noted that the concentrations of the Ti and Ni ions were similar in the pH 1.68 solutions, but differed by one to two orders of magnitude in the other solutions (Table 2).

The dissolutions of both Ni and Ti in the acidic organic buffer solutions (pH 1.68–4.01) can be explained by the fact that complex salts are produced when these ions interact with potassium tetraoxalate, tartrate, and phthalate [84]. Titanium is considered to have satisfactory resistance to chloride-containing environments, with the pitting potentials of Ni and Ti in 0.1% NaCl at 25 °C being +0.28 and +12.0 V, respectively, which is a difference of exactly two orders of magnitude [84,85].

However, according to literature data, Ni ions are released into the solution after corrosion, while Ti remains on the surface and reacts with the dissolved O to form an oxide layer [24]. Previous reports also suggested the presence of a subsurface Ni_3_Ti layer and pure Ni particles measuring 10–100 nm between the surface oxide layer and the bulk alloy, attributable to the formation of a thick TiO_2_ layer [25,42,62]. The reaction of O with Ti is more thermodynamically favorable than that of O with Ni. A thin layer of titanium oxide thus begins to form spontaneously on the NiTi surface when it is exposed to an O-containing environment. This oxidation changes the crystalline structure of the NiTi surface and forms a Ni-enriched sublayer, which contains vacancies where the Ti atoms were knocked out from their position in the crystal lattice [92]. The Ni atoms can be released from their bonds with Ti (Ni–Ti) at close to room temperature, with the atoms subsequently occurring as crystalline defects—interstitial atoms in the structure of the surface oxide. This oxide is typically non-stoichiometric due to its O deficiency. The sites of the missing O atoms also constitute structural vacancies, which allows the diffusion of Ni through the oxide layer even at a low temperature due to the smaller size of the Ni atoms compared with the Ti and O atoms. Thus, Ni could dissolve from the surface of the NiTi sample as well as from the subsurface layers, while Ti remains in the sample and forms a protective oxide surface layer that thickens with time, as can also be observed from Figure 8 and Figure 10 [24,25,33,42].

We also found evidence of the selective dissolution of metals [23,93]. In a previous study, Ti was observed to leach only into acidic solutions and Ni into both acidic and neutral solutions, and the surface damage caused by the leaching was subsequently repaired by titanium oxide formation after Ni etching [93]. In another study, Ti ions were observed to only be released in artificial saliva at pH 2.5, and not in less acidic or neutral solutions [23].

Our observation of the release of Ti ions into neutral solutions was thus unexpected, although the Ti ion concentration was lower than that of the Ni ions for all the investigated solutions (Figure 10, Figure 11a; Table 2). However, as Ti ion release was only observed for the non-mechanically treated samples (Table 2), the release was apparently controlled by the NiTi nanostructures. We speculate that the fine grains in the areas with a high density of defects completely broke down with the washing-out of Ni, resulting in Ti release. Although Ti is not considered to be harmful to the human body, even at much higher concentrations, the nanostructure has a double effect on the corrosion resistance of nitinol.

This effect was also confirmed by previous reports. For example, Amirhanova N.A. et al. [94] observed an ambiguous effect of the nanostructures of NiTi with grain diameters less than 10 nm. The corrosion resistance was found to be substantially lower in HCl solution compared with the microstructured NiTi due to the larger number of grain boundaries and structural defects. However, the alloy was more corrosion resistant in NaCl solutions. In other studies [91,95,96,97], no difference was found between the corrosion processes of micro- and nanostructured NiTi. However, nanostructured Ti was found to exhibit reduced corrosion resistance [98]. However, a positive influence on the mechanical properties of the materials was noted in these studies.

The observed effects of the treatment methods used in this study on the ion release agree closely with previous reports on microstructural nitinol, suggesting that heat treatment significantly reduces the corrosion resistance due to the mixed formation of surface layers containing titanium oxide and Ni oxide [16,19,42,44,45]. The negative effects of the heat treatment (Figure 4A,B; Figure 10) may also be attributed to the drawing back and recrystallisation of the outer cold-worked layer formed during the wire fabrication process. Thus, the behavior of the nanostructured nitinol after heat treatment does not differ from the microstructural behavior.

However, the effects of the mechanical treatment were found to be greater (Figure 7; Table 2) than those previously reported [16,26,27,45]. No Ti release from the mechanically treated samples was observed in the solutions with pH 4.01–6.31, in contrast to the observation in the pH 1.68 solution and for the other samples. Based on the present observation of a significant reduction in the Ni ion release (and generally low concentration in solutions) and no Ti ion release from the mechanically treated NiTi samples (and no metal release in artificial plasma), as well as the structural analysis of mechanically treated nitinol samples after exposure to these solutions (Figure 7, Figure 8; Table 2), we conclude that a strong homogenous protective layer of titanium oxide formed on the surface of the samples, creating a barrier to Ni dissolution. That is, a combination of nanostructuring and subsequent mechanical treatment significantly positively affects the corrosion resistance of NiTi.

Notably, the above observations occurred under static conditions, which may not be representative of physiological conditions. The formation of a heterogenous mixed layer of Ti and Ni oxides after annealing, which is a necessary process for obtaining a finished NiTi device, would expose live tissues to direct contact with toxic Ni and also allow for Ni diffusion into the surrounding environment.

The use of nanostructured NiTi for medical implants and devices thus remains prospective compared with microstructured NiTi, although the formation of a corrosion-resistant and biocompatible surface layer is a desirable feature.

## 5. Conclusions

Less Ni was released from the nanostructured NiTi samples investigated in this study in solutions with different acidity levels than from microstructured NiTi. Mechanical treatment was found to increase the corrosion resistance of NiTi by two- to three-fold. Heat treatment reduced the corrosion resistance of the samples, and the nanostructures apparently induced the release of Ti ions in solutions with any acidity. The highest concentration of metals was observed in solutions with the highest acid concentration. Smaller amounts of metal were found to be released into neutral saline, and the lowest amounts into weakly acidic solutions. Metal dissolution did not occur in an alkaline environment and artificial plasma. The metal concentration in solutions increased over time, but the rates of increase varied for different samples and solutions.

Significantly less Ni and no Ti was released from the mechanically treated samples in neutral and weakly acidic solutions, whereas no metal was released whatsoever in artificial plasma. This implies the formation of a strong and homogenous protective titanium oxide layer that prevents Ni dissolution. This layer afforded the high corrosion resistance of the mechanically treated nanostructured NiTi samples.

The surface compositions of the as-received and the unpolished annealed wires were observed to be similar, consisting of interspersed areas of carbon- and titanium-oxide-rich spots that reached depths of 3 µm. The long exposure of the wire samples to different solutions did not significantly change the surface composition. Mechanical treatment of the wire surface resulted in the formation of a thin uniform titanium oxide layer, and reduced the surface roughness and defects, although it did not entirely remove Ni from the surface. A Ni-free surface with a protective 25 nm-thick titanium oxide film resulted from soaking mechanically treated samples of the NiTi wire in a saline solution for two years under static conditions. Such conditions are, however, impractical in medical applications.

Hence, the medical application of nanostructured NiTi, such as for the production of medical devices and implants such as stents, remains prospective compared to microstructured NiTi. However, the formation of a corrosion-resistant and biocompatible surface layer is a desirable feature of nanostructured NiTi.

## Figures and Tables

**Table 1:** pH and composition of the solutions used for immersion tests.

Solution Number	pH	Composition
1	1.68	Potassium tetraoxalate KH_3_C_4_O_8_·2H_2_O, 0.05 M
2	3.56	Acid potassium tartrate C_4_H_5_O_6_K, 0.025 M
3	4.01	Acid potassium phthalate C_8_H_5_O_4_K, 0.05 M
4	6.31	Sodium chloride NaCl, 0.9 wt %
5	9.18	Acid sodium tetraborate Na_2_B_4_O_7_·10H_2_O, 0.05 M
6	7.36	Artificial plasma: NaCl (92.3 mM), NaHCO_3_ (26.3 mM), K_2_HPO_4_ (0.9 mM), KCl (2.7 mM), NaH_2_PO_4_ (0.22 mM), CaCl_2_ (2.5 mM), MgSO_4_·7H_2_O (0.82 mM), Na_2_SO_4_ (1.48 mM), d-glucose C_6_H_12_O_6_ (5.55 mM) [16,17,20,23,26,27,29,30,46,49,78]
7	1.56	HCl (aq.), 0.0275 M

**Table 2:** Metal concentrations in various solutions measured by inductively coupled plasma atomic emission spectroscopy (ICP-AES) for various immersion times and samples types.

Solution Number	Time (Days)	Ion Concentration in Solution (mg/L)							
		As-Received Sample (1)		Annealed Sample (2)		Mechanically Treated Sample (3)		Annealed Mechanically Treated Sample (4)	
		Ti	Ni	Ti	Ni	Ti	Ni	Ti	Ni
1	0	0.00	0.00	0.00	0.00	0.000	0.000	0.000	0.000
	10	1.30	2.13	4.00	6.20	0.470	0.710	0.317	0.245
	25	1.90	2.97	6.50	9.00	0.640	0.940	0.467	0.784
	45	2.30	3.67	9.08	9.97	0.750	1.150	0.556	0.958
	60	3.05	4.16	9.81	10.44	0.810	1.250	0.614	1.087
	75	3.14	4.26	9.86	10.55	0.860	1.300	0.656	1.182
	287	3.15	4.29	10.01	10.70	1.070	1.550	1.284	1.860
	754	9.60	13.39	20.70	24.60	3.340	4.550	4.559	5.898
2	0	0.00	0.00	0.00	0.00	0.00	0.00	0.00	0.00
	10	-	0.05	-	0.10	-	0.03	-	0.010
	25	-	0.18	-	0.40	-	0.07	-	0.058
	45	-	0.38	0.08	0.61	-	0.11	-	0.092
	60	0.04	0.44	0.09	0.67	-	0.13	-	0.113
	75	0.06	0.47	0.09	0.87	-	0.14	-	0.127
	287	0.12	1.05	0.16	1.59	-	0.19	-	0.228
	754	0.32	1.34	0.48	2.19	0.09	0.32	0.11	0.415
3	0	0.00	0.00	0.00	0.00	0.00	0.00	0.00	0.00
	10	-	0.03	-	0.04	-	0.01	-	0.003
	25	-	0.06	-	0.07	-	0.02	-	0.017
	45	-	0.07	-	0.14	-	0.02	-	0.017
	60	-	0.09	-	0.14	-	0.03	-	0.026
	75	-	0.10	-	0.18	-	0.03	-	0.027
	287	-	0.41	-	0.57	-	0.10	-	0.120
	754	0.01	0.50	0.01	0.63	-	0.14	-	0.181
4	0	0.00	0.00	0.00	0.00	0.00	0.00	0.00	0.00
	10	-	0.50	0.01	0.88	-	0.04	-	0.014
	25	-	0.76	0.05	1.33	-	0.12	-	0.100
	45	0.01	0.94	0.09	1.55	-	0.15	-	0.125
	60	0.03	0.95	0.10	1.56	-	0.17	-	0.148
	75	0.04	0.97	0.12	1.59	-	0.19	-	0.173
	287	0.08	1.20	0.17	2.08	-	0.24	-	0.288
	764	0.12	1.28	0.19	2.17	-	0.39	-	0.506
7	0	0.00	0.00	0.00	0.00	0.00	0.00	0.00	0.00
	10	0.515	1.94	no	no	no	no	no	no

Note: -, a concentration less than 0.0066 mg/L; no, no data.

**Table 3:** Diameters of each type of NiTi wire sample before and after immersion for two years in solutions with various pH values.

Diameter Before Immersion (µm)	Diameter After Immersion (µm)	Sample	pH
280.00	253.74	As-received	1.68
280.00	275.59	As-received	3.56
280.00	277.14	As-received	4.01
280.00	275.16	As-received	6.31
280.00	280.00	As-received	9.18
280.00	252.46	Annealed	1.68
270.00	257.00	Mechanically treated	1.68
280.00	270.03	As-received	1.5 (HCl)

**Table 4:** Surface composition of mechanically treated NiTi samples after immersion in various solutions for two years.

Solution pH	1.68	3.56	4.01	6.31/7.36	9.18
Oxide layer thickness (nm)	8–13	13–17	15–20	23–28	~10
Nickel surface concentration (atomic %)	8	5	3	0	3
Depth of nickel etching (nm)	10	10	10	20	10
Maximum oxygen surface concentration (atomic %)	~60–70 atomic.% at a depth of 2.5–7 nm				

**Table 5:** Nickel concentrations in various solutions for various immersion times and nitinol samples types reported in the literature.

Solution		Time (Days)	Ni Concentration in Solution (mg/L)		Reference
			As-Received Sample (Commercial)	Mechanically Treated and/or Electropolished Sample	
0.9% NaCl		15	0.005–1.000		[25]
		30	0.010–1.480		
		60	0.020–1.620		
		120	0.025–1.760		
		180	0.030–1.800		
Hank’s physiological solution (pH 7.4)		1	0.200	0.200	[26]
		2	0.300	0.220	
		6	0.350–0.400	0.250	
		9	0.500–0.850	0.250	
		14	0.550–0.900	0.250	
		18	0.600–1.050	0.250	
		1		1.300	[27]
Simulated body fluid (SBF) (pH 7.4)		14		0.195	[28]
		35		0.205	
		70		0.500	
		30		0.290	[29]
		35	0.950		[30]
(cell-free) Culture medium (pH 7.4)		2	0.152		[32]
		2	118.930		[33]
		3		0.081	[22]
		6		0.176	
Artificial saliva	pH 2.5	1	0.255–1.275		[23]
		3	1.530–4.080		
		7	2.040–5.610		
		14	4.080–10.200		
		28	4.080–15.300		
	pH 3.75	1	0.128–0.893		
		3	0.255–1.020		
		7	0.255–1.275		
		14	0.255–1.785		
		28	0.383–2.550		
	pH 6.25	1	0.038–0.191		
		3	0.153–0.561		
		7	0.153–0.714		
		14	0.153–0.714		
		28	0.153–0.765		
0.9% NaCl		2		0.013	[24]
		5		0.020	
		8		0.050	
		12		0.065	
		30		0.120	
